# Choosing Your Endoscopist: A Retrospective Single-Centre Cohort Study

**DOI:** 10.7759/cureus.67403

**Published:** 2024-08-21

**Authors:** Jun Guang Kendric Tan, Nicole Lee Chui Hew, Mary Theophilus, Ruwan Wijesuriya

**Affiliations:** 1 General Surgery, St. John of God Midland Public and Private Hospitals, Perth, AUS

**Keywords:** quality improvement, colonoscopy, surveillance, colorectal cancer, adenoma detection rate

## Abstract

Background

Colorectal cancer is one of the most common internal malignancies affecting Australians, and colonoscopy is widely accepted as a part of comprehensive large bowel assessment. Different specialties perform colonoscopies, most commonly general surgeons and gastroenterologists. Analysing performance outcomes against benchmarks allows insight into inter-specialty differences and enables the improvement of overall service provision and quality.

Methods

We performed a retrospective single-centre cohort study on 2086 patients undergoing colonoscopies by seven surgeons (S) and nine gastroenterologists (G) between July 2021 and June 2023. Primary outcomes were comparative caecal intubation rates (CIR), photo documentation rates (PDR), documented withdrawal rates (DWR), withdrawal times (WT), and adenoma detection rates (ADR). Secondary outcomes characterised adenoma frequency, optimal WT, and indications for colonoscopies.

Results

We found significant differences in CIR (S: 94.9%, 990/1043; G: 99%, 1033/1043, P<0.01), PDR (S: 95.9%, 949/990; G: 99.1%, 1024/1033, P<0.01), DWR (S: 17.4%, 181/1043; G: 87.3%, 911/1043, P<0.01), WT >6 minutes (S: 82.3%, 149/181; G: 97.8%, 891/911, P<0.01), and ADR (S: 37.9%, 193/509; G: 59.7%, 421/705, P<0.01). Subgroup analysis revealed adenoma frequency peaked at 50-70 years old and optimal WT was ≥9 minutes. We demonstrated surgeons mainly perform colonoscopies for diverticulitis surveillance, abnormal imaging, post-cancer resections, and rectal bleeding, but gastroenterologists predominantly investigate bowel symptoms, polyp surveillance, positive faecal occult blood test, and anaemia.

Conclusion

Despite both specialties surpassing national standards in CIR and ADR, there were significant differences in performance indicators. We believe ADR differences could be explained by different indications specialties perform colonoscopies for. Increasing WT ≥9 minutes could improve ADR, and education on the usage of withdrawal timer on endoscopes will improve DWR.

## Introduction

Colorectal cancer (CRC) is the second most common internal malignancy affecting Australians [1]. Incidence peaked in 2007 with 65.2 cases per 100,000 persons, before dropping to 48.9 cases per 100,000 persons in 2022 [1]. This can be attributed to robust national surveillance programs and well-established surveillance schedules. CRC's well-documented biological pathway of adenoma-cancer sequence means that appropriately timed colonoscopies can reduce CRC malignancy rates by the early detection and removal of pre-malignant adenomas, thus preventing the progression to malignancy [2]. High-quality colonoscopies are the current gold standard for large bowel assessment, allowing for direct visualisation, diagnosis, biopsies, and interventions such as polypectomy.

Clinical guidelines have been released by all major endoscopic societies: the American Society for Gastrointestinal Endoscopy (ASGE) [3], the European Society of Gastrointestinal Endoscopy (ESGE) [4], the UK Joint Advisory Group on GI Endoscopy (JAG) [5], and Australia's National Health and Medical Research Council (NHMRC) [6]. There are well-established quality indicators for colonoscopies which are similar across all societies, with the main five indices being adequate bowel preparation, caecal intubation rate (CIR), adenoma detection rate (ADR), average withdrawal time (WT), and complications. Of the many indices, ADR is the most established indicator and independently predicts CRC diagnosis after colonoscopy [7,8]. Low ADRs and poor-quality colonoscopies are not only associated with immediate complications and failure to detect pre-malignant lesions but also inappropriately prolonged surveillance intervals which negatively impact post-colonoscopy CRC rates [3].

Australia has a unique geographical layout, with about seven million individuals, or 28% of the Australian population, living in rural and remote areas [9]. This places them outside the reach of traditionally metropolitan-based healthcare systems. A study by Choi et al. revealed an endoscopist every 0.33-62.05 km^2^ in capital cities, increasing to every 23,382-267,780 km^2^ in rural areas [10]. The majority of colonoscopies in rural settings are performed by rural surgeons, as gastroenterology tends to be a visiting service. Given the scarcity of gastroenterologists in the outback, general surgeons provide an essential service to our large rural and remote population. It is therefore critical to ensure that all specialties offer equally robust services.

There has been increasing interest in the impact of the endoscopist's specialty on colonoscopic quality, particularly between surgeons and gastroenterologists. Some centres have found surgeons to have generally poor-quality metrics and outcomes [11], while others revealed no difference in ADR or post-colonoscopy CRC [12]. There can also be large variations in ADR within proceduralists of the same specialty, with differences attributed to key quality metrics such as mean WT [13]. This begs the question: "Are outcomes truly dependent on the specialty of the proceduralist or adherence to guidelines and individual performance?".

## Materials and methods

Study design

**Figure 1:**
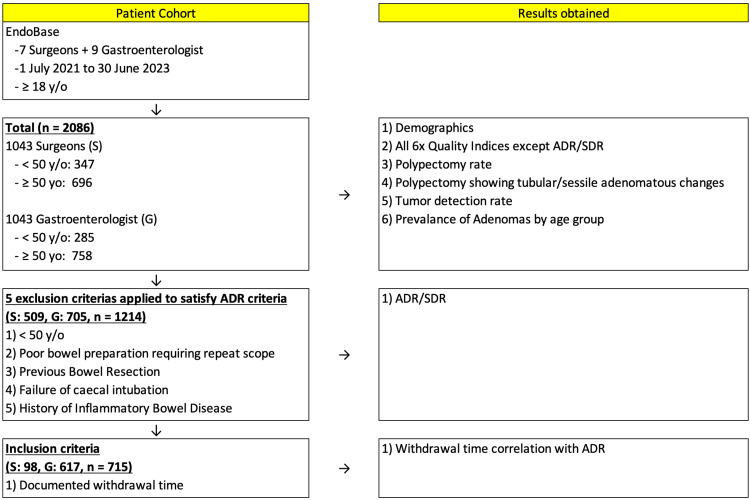
Study design y/o: years old; S: surgeons; G: gastroenterologist; ADR: adenoma detection rate; SDR: serrated polyp detection rate

This retrospective cohort study was undertaken at a 300-bed secondary hospital in Western Australia, Perth. The study design is outlined in Figure 1.

Two thousand and eighty-six consecutive patients undergoing colonoscopy by seven surgeons and nine gastroenterologists between July 1, 2021, and June 31, 2023, were identified from the endoscopy database (Olympus Endobase). The median age of all patients was 59 (19-93), and the mean age of patients above the age of 50 was 65 (50-93). NHMRC quality indicators were derived from these colonoscopy reports and analysed. To assess true ADR, exclusion criteria were applied to identify colonoscopies performed for patients older than 50 years old, who have intact colons, and who have no previous suspicion of inflammatory bowel disease. These patients were used to derive values for ADR and serrated polyp detection rates (SDR). The correlation of WT and ADR was calculated when available. Other exclusion criteria included incomplete entries which would not allow for statistical analysis. Information about included cases were obtained from electronic medical records. All endoscopists are either certified by the Gastroenterological Society of Australia (GESA) or supervised by a GESA-certified endoscopist.

Data collection

Data was collected by two investigators and included patient demographics, endoscopist specialty, trainee involvement, procedure duration and indication, quality of bowel preparation, documented caecal intubation, photo documentation of appendix orifice/ileocaecal valve/terminal ileum, procedural findings, histopathology, and complications. Trainees were either registered surgical or gastroenterology trainees under the Royal Australasian College of Surgeons or Royal Australasian College of Physicians, who have not attained GESA certification. When trainees were involved, there was direct supervision by consultant surgeons or physicians with appropriate interventions at technically challenging steps.

Statistical analysis

Comparative statistics were performed with a two-tailed Fisher's exact test. A p-value of <0.05 was considered statistically significant.

Ethics

Local ethics approval was obtained through the St. John of God Health Care Human Research Ethics Committee (approval number: 2080).

## Results

Primary outcome 

Although both specialties surpassed minimal ADR, gastroenterologists outperformed surgeons in six out of eight aspects. Clearly documented indications had the best performance. Bowel preparation rates were excellent, with only 21 patients requiring repeat colonoscopy. Caecal intubation for both specialties met the minimal cutoff of >90%, but there was a significant difference of 4.1% (n=43), with surgeons intubating 94.9% (990/1043) and gastroenterologists intubating 99% (1033/1043, P<0.01) of the time. Photo documentation was lacking for surgeons, with 95.9% (949/990) documentation, 3.2% (n=75) less than gastroenterologists. DWR were poorly done for surgeons at 17.4% (181/1043) when gastroenterologists had superior rates of 87.9% (911/1043, P<0.01). ADR were both above the target rate of 25%. However, gastroenterologists' ADR was almost 1.5 times that of surgeons. SDR for surgeons were 7.3% (37/509), falling below NHMRC standards. However, this is a relatively new quality indicator and not as robust as ADR. Top causes for failed caecal intubation were colonic redundancy and diverticular associated stricturing (S: 64.1%, 34/53; G: 50%, 5/10), poor bowel prep (S: 22.6%, 12/53; G: 40%, 4/10), and obstructing masses (S: 13.2%, 7/53; G: 10%, 1/10). PDR were 95.9% (949/990) and 99.1% (1024/1033), respectively, leaving room for improvement. Comparative figures of colonoscopies performed by surgeons and gastroenterologists against quality indices set out by the 2018 NHMRC guidelines [6] are outlined in Table 1. 

**Table 1:** 2018 NHMRC quality indices NHMRC: National Health and Medical Research Council

NHMRC quality indicators	NHMRC standard	General surgery	Gastroenterology	P-value
1. Documented indications	100%	99.5%	99.9%	0.22
		1038/1043	1042/1043	
2. Poor bowel preparation rate	<10%	1.2%	0.9%	0.66
		12/1043	9/1043	
3. Caecal intubation rate	>90%	94.9%	99%	<0.01
		990/1043	1033/1043	
4. Photo documentation rate	100%	95.9%	99.1%	<0.01
		949/990	1024/1033	
5.i. Documented withdrawal rate	100%	17.4%	87.3%	<0.01
		181/1043	911/1043	
5.ii. Withdrawal time ≥6 mins	100%	82.3%	97.8%	<0.01
		149/181	891/911	
6. Adenoma detection rate ≥50 y/o	>25%	37.9%	59.7%	<0.01
		193/509	421/705	
7. Serrated polyp detection rate ≥50 y/o	>10%	7.3%	16%	<0.01
		37/509	113/705	
8. Perforation rate	<0.1%	0%	0%	1.00
		0/1043	0/1043	

Secondary outcomes

**Figure 2:**
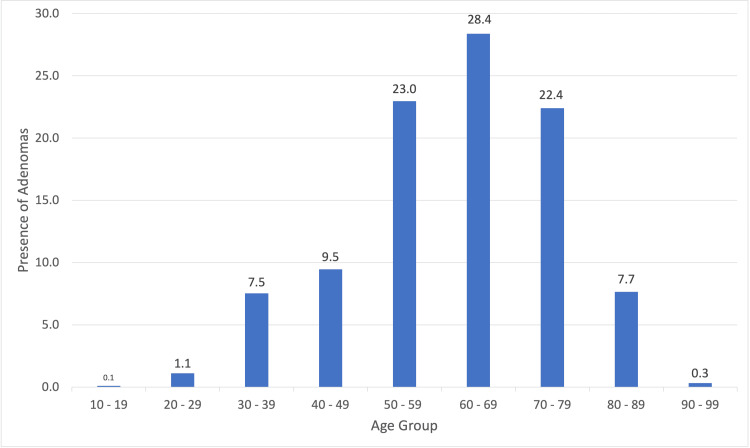
Prevalence of adenomas by age group

The overall polypectomy rate for surgeons was 46% (479/1043), while for gastroenterologists, it was 67% (696/1043, P<0.01). More importantly, the rate of polyps with adenomatous changes was 69% (331/479) for surgeons and 80% (557/696, P<0.01) for gastroenterologists. Overall tumor detection was 1.8% (19/1043) for surgeons and 1.1% (11/1043, P=0.20) for gastroenterologists. We saw a peak in adenoma frequency after 50 years old, jumping from about 9.5% (84/294) at ages 40-49 to 23% (204/468) at ages 50-59, highlighting the increasing frequency of early bowel cancer. This peak in adenoma frequency continues into the ages of 60-69 at 28.4% (252/478), before gradually decreasing in frequency to 22.4% (199/366) at ages 70-79 and 7.7% (68/130) for 80-89. This is demonstrated in Figure 2.

Correlation between ADR and WT

**Figure 3:**
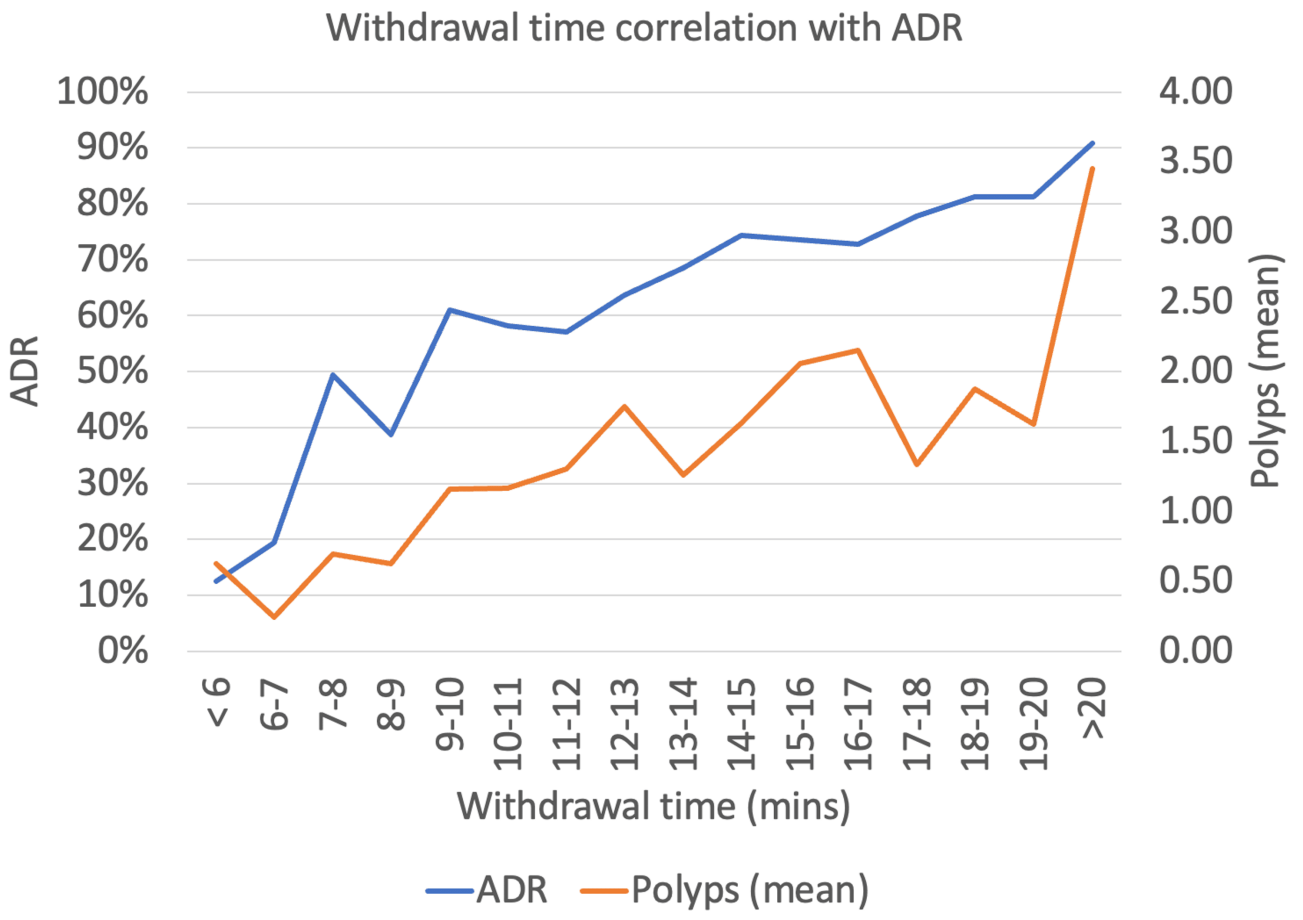
Withdrawal time correlation with ADR and mean polypectomy count ADR: adenoma detection rate

There is a linear relationship between WT from eight minutes (ADR: 39%) to ≥20 minutes (ADR: 91%). Figure 3 demonstrates that ADR is only consistently ≥25% when WT is ≥7 minutes and is ≥57% when WT is ≥9 minutes. The mean polypectomy count also increases with WT at 0.55 with WT <9 minutes, 1.33 with WT 9-14 minutes, and 2.02 with WT >14 minutes.

Spectrum of indications for colonoscopy

**Figure 4:**
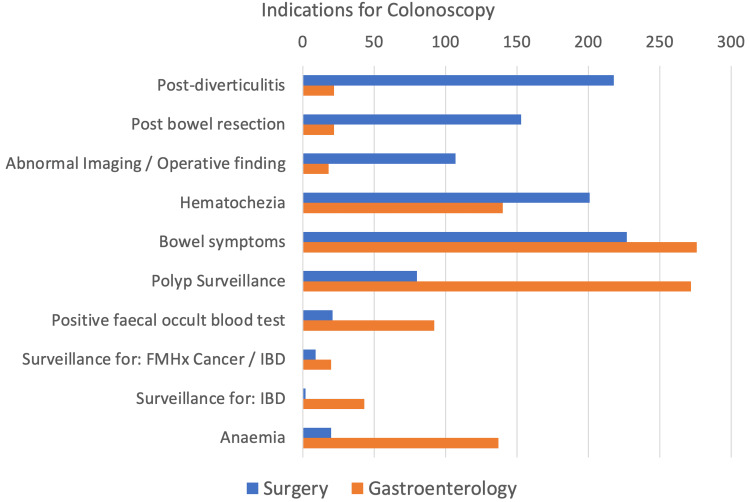
Indications for colonoscopy Bowel symptoms range from alternating bowel habits, abdominal pain, bloating, flatulence, and weight loss FMHx: family history; IBD: inflammatory bowel disease

We see surgeons frequently survey cancer and diverticular disease, while gastroenterologists investigate mainly bowel symptoms, iron deficiency anaemia, inflammatory bowel disease, and polyp screening. This is demonstrated in Figure 4.

## Discussion

There are 11 main quality markers outlined in the 2018 NHMRC clinical guidelines [6] for surveillance colonoscopy. Here, we will discuss four main markers and analyse a few trends highlighted by our study.

ADR

ADR is the proportion of colonoscopies in which adenomas are detected. As most CRC develop via the adenoma-carcinoma sequence [2], ADR is generally considered the most reliable marker for post-colonoscopy CRC rates and widely used as a marker for colonoscopy quality [14]. A low ADR has been associated with higher rates of post-colonoscopy CRC [8], and a 3% decrease in risk for post-colonoscopy CRC is associated with every 1% increase in ADR [15].

NHMRC, ASGE, and ESGE recommend an ADR of ≥25% and JAG of ≥15%. In our series, the ADR by surgeons was 37.9% (193/509), and that of gastroenterologists was 59.7% (421/705). Although both specialties met the recommended threshold, the ADR for gastroenterologists is almost 50% more than that of surgeons. This can be explained by different patient demographics and colonoscopic indications between the two specialties. Most surgeons perform very little "true screening colonoscopies" but rather investigate incidental radiological or intra-operative findings. In addition, surgeons perform the majority of post-cancer resection surveillance colonoscopies, where most polyps have been removed prior. This is unlike the gastroenterologists who perform almost twice as many colonoscopies for polyp surveillance, naturally raising the ADR.

CIR and photo documentation

Up to 15% of all CRC are found in the caecum or appendix [16]. These can easily be missed if direct visualisation of the entire colon is not achieved. Formal photo documentation involves visualising the ileocaecal valve, appendices orifice, tri-radiate caecal folds, or terminal ileum. This removes subjective interpretation and ensures active effort is taken to reach the caecum, ensuring a complete examination of the large bowel. ASGE, ESGE, JAG, and NHMRC all recommend "auditable photo documentation of completion," aiming for rates ≥90%. In our study, surgeons' CIR was 94.9% (990/1043) and gastroenterologists' CIR was 99% (1033/1043).

The majority of failed caecal intubation were due to colonic redundancy and diverticular associated stricturing (S: 64.1%, 34/53; G: 50%, 5/10). In these patients, if the risk of potential colonic perforation is deemed unacceptably high, colonoscopy will be abandoned for second-line investigations such as computed tomography colonography. The next most common reason is poor bowel preparation (S: 22.6%, 12/53; G: 40%, 4/10) where a colonic luminal examination is not adequate to rule out significant lesions. Patients will be offered extended bowel preparation and a repeat colonoscopy offered in a timely fashion. Finally, obstructing masses (S: 13.2%, 7/53; G: 10%, 1/10) are the final reason for failed caecal intubation. These are frequently suspicious for malignancy. In our centre, staging investigations and colorectal surgical input are promptly arranged with a view to a multidisciplinary team management approach.

Bowel preparation

Good-quality colonoscopies require excellent visualisation of the colonic mucosa. There has been much improvement in the technical quality of video in recent years, but the crucial prerequisite is still high-quality bowel preparation. This is especially true with the identification of sessile polyps, which have very subtle differences from the surrounding mucosa. Poor preparation has been associated with higher rates of incomplete colonoscopies [17], prolonged procedure times [18], and reduced yield [19]. There are multiple regimens for bowel preparation. The preferred preparation at our institution is split bisacodyl and polyethylene glycol (PEG) preparations as they cause fewer electrolyte disturbances and are suitable for patients with comorbidities and the elderly. PEG preparations, if administrated appropriately, can have very good outcomes, demonstrated by very low poor bowel preparation rates at 1% (21/2086) at our institution. Bowel preparation regiments are uniform for both surgical and gastroenterology cohorts which explains the lack of difference between both specialties.

WT

Visualisation of the colonic wall is mostly performed on withdrawal from the caecum to the rectum. A longer WT has been associated with higher ADR. Several factors can explain the superior ADR. These include increased duration of washing and suctioning the colonic mucosa to ensure optimal views, meticulous examination of all colonic folds, double passes of each flexure, and employing positional changes [20]. In our series, 98.9% (707/715) of colonoscopies fulfilling ADR criteria had WT ≥6 minutes. Of note, ADR was consistently ≥57% when WT is ≥9 minutes. This likely reflects the optimal WT needed to visually inspect the colon for neoplasia. Hence, we recommend a minimal WT of nine minutes for high-quality colonoscopies.

One must note the inverse holds true as well, that is, polyp detection with associated polypectomy increases WT. We have shown that increasing WT leads to both increased ADR and polypectomy rates. This reflects increased fidelity as one is both sampling more polyps and more accurately identifying macroscopically suspicious-appearing lesions. Extended durations of >20 minutes are likely from patients with polyposis syndromes requiring extended duration to perform polypectomies.

We did note WT was poorly documented by surgeons (17.4%, 181/1043) compared to gastroenterologists (87.3%, 911/1043). We believe this is due to poor usage of the withdrawal timer functionality on our endoscopes. If used appropriately, WT is automatically registered.

Indications for colonoscopy per specialty

Surgeons perform the majority of post-diverticulitis and post-cancer resection surveillance colonoscopies as patients would have been admitted under a surgical team for prior management. Surgeons also perform surveillance colonoscopies for abnormal imaging or intra-operative findings. These are usually incidental findings on computed tomography scans, be it abnormal bowel wall thickening or unusual inflammatory changes. Both specialties investigate per-rectal (PR) bleeding with similar frequency. If the initial PR bleed was severe enough to be admitted under general surgery for management, follow-up surveillance colonoscopies are usually performed by surgeons. However, referrals from the community can be referred to either surgeons or gastroenterologists depending on the general practitioner's assessment.

Gastroenterologists investigate largely non-specific bowel symptoms, e.g., abdominal pain, altered bowel habits, bloating, weight loss, and perianal disease. These referrals are usually from the community and reflect the referral patterns of general practitioners. This is the same for polyp surveillance where there are clear guidelines on surveillance intervals. The responsibility to organise subsequent colonoscopies is often handed back to the general practitioner who actions subsequent referrals. Inflammatory bowel disease and iron deficiency anaemia are usually managed medically; hence, gastroenterologists perform more follow-up colonoscopies in that domain. Endoscopic mucosal resection requires further training in endoscopy techniques and is mainly performed by gastroenterologists.

Possible limitations of our study include not controlling for different disease processes. Our primary endpoint was to measure overall performance between two specialties based on quality indices set out by the 2018 NHMRC guidelines. Hence, stratification by indications and pathologies was not performed. Instead, our quality indices are reflective of the true patient population rather than individual disease processes and thus more representative of real-life practice. Another limitation is our use of summary-level data instead of individual patient-level data. Patient risk factors for colorectal neoplasia such as family history, cigarette smoking, and obesity were not available, and it is uncertain if they will have an impact on ADR between the two groups.

## Conclusions

The debate of colonoscopic outcomes between surgeons and gastroenterologists fails to fully appreciate their vastly different scopes of practice. We have shown both specialties perform colonoscopies for very different indications and this likely has a negative impact on surgery's ADR. Despite this, both surgeons and gastroenterologists exceed the benchmark standards in terms of ADR and CIR suggested by NHMRC, ASGE, ESGE, and JAG. There is however room for improvement in WT and photo documentation, and education on the usage of withdrawal timer and photo functionality on endoscopes should improve these metrics. In addition, simple measures such as increasing WT ≥9 minutes could improve ADR.

Our study reinforces current knowledge that colonoscopies performed by accredited clinicians in both general surgery and gastroenterology are of high quality and auditable to international standards. A repeat study after the implementation of our suggested measures should yield improved outcomes.

## References

[1] (2023). Cancer data in Australia. https://www.aihw.gov.au/reports/cancer/cancer-data-in-australia/contents/cancer-rankings-data-visualisation.

[2] Morson B (1974). President's address. The polyp-cancer sequence in the large bowel. Proc R Soc Med.

[3] Rex DK, Schoenfeld PS, Cohen J (2015). Quality indicators for colonoscopy. Gastrointest Endosc.

[4] Kaminski MF, Thomas-Gibson S, Bugajski M (2017). Performance measures for lower gastrointestinal endoscopy: a European Society of Gastrointestinal Endoscopy (ESGE) quality improvement initiative. Endoscopy.

[5] Rees CJ, Thomas Gibson S, Rutter MD, Baragwanath P, Pullan R, Feeney M, Haslam N (2016). UK key performance indicators and quality assurance standards for colonoscopy. Gut.

[6] (2018). Clinical practice guidelines for surveillance colonoscopy. https://www.cancer.org.au/clinical-guidelines/bowel-cancer/surveillance-colonoscopy.

[7] Sekiguchi M, Falkén Y, Matsuda T, Saito Y, Hultcrantz R (2022). Colonoscopy quality and endoscopist factors: what are the required endoscopist conditions for high-quality colonoscopy to reduce colorectal cancer incidence and mortality?. Mini-invasive Surgery.

[8] Kaminski MF, Regula J, Kraszewska E (2010). Quality indicators for colonoscopy and the risk of interval cancer. N Engl J Med.

[9] (2024). Rural and remote health. https://www.aihw.gov.au/reports/rural-remote-australians/rural-and-remote-health.

[10] Choi MS, van der Mark MA, Hung K (2022). The distribution and composition of colonoscopy providers in Australia. Cureus.

[11] Mazurek M, Murray A, Heitman SJ (2022). Association between endoscopist specialty and colonoscopy quality: a systematic review and meta-analysis. Clin Gastroenterol Hepatol.

[12] Lee AH, Lojanapiwat N, Balakrishnan V, Chandra R (2018). Is there a difference in adenoma detection rates between gastroenterologists and surgeons?. World J Gastrointest Endosc.

[13] Barclay RL, Vicari JJ, Doughty AS, Johanson JF, Greenlaw RL (2006). Colonoscopic withdrawal times and adenoma detection during screening colonoscopy. N Engl J Med.

[14] Millan MS, Gross P, Manilich E, Church JM (2008). Adenoma detection rate: the real indicator of quality in colonoscopy. Dis Colon Rectum.

[15] Corley DA, Jensen CD, Marks AR (2014). Adenoma detection rate and risk of colorectal cancer and death. N Engl J Med.

[16] Rees CJ, Bevan R, Zimmermann-Fraedrich K (2016). Expert opinions and scientific evidence for colonoscopy key performance indicators. Gut.

[17] Hendry PO, Jenkins JT, Diament RH (2007). The impact of poor bowel preparation on colonoscopy: a prospective single centre study of 10,571 colonoscopies. Colorectal Dis.

[18] Bernstein C, Thorn M, Monsees K, Spell R, O'Connor JB (2005). A prospective study of factors that determine cecal intubation time at colonoscopy. Gastrointest Endosc.

[19] Jang JY, Chun HJ (2014). Bowel preparations as quality indicators for colonoscopy. World J Gastroenterol.

[20] Lee RH, Tang RS, Muthusamy VR (2011). Quality of colonoscopy withdrawal technique and variability in adenoma detection rates (with videos). Gastrointest Endosc.

